# Modulation of CT1 Function: From Klotho Protein to Ammonia and Beyond

**DOI:** 10.3389/fnut.2021.660021

**Published:** 2021-05-10

**Authors:** Sergej M. Ostojic

**Affiliations:** FSPE Applied Bioenergetics Lab, University of Novi Sad, Novi Sad, Serbia

**Keywords:** creatine, SCL6A8, starvation, Klotho protein, mTOR, hyperammonemia

## Introduction

The creatine transporter (CT1 or SLC6A8) is a sodium- and chloride-dependent multi-pass membrane protein required for the cellular uptake of creatine, a key high-energy phosphate-storage molecule. This substrate-specific carrier (Table 1) is located across the plasma membrane of various energy-demanding cells and organs, including the brain, skeletal muscle and myocardium, gastrointestinal tract, kidney and urinary bladder, male and female organs, skin, bone marrow, and granulocytes. CT1 defect or malfunction is characterized by a severe depletion of the intracellular creatine pool, accompanied by intellectual disability, seizures, and movement and behavior disorders (1, 2). Transferring creatine through biomembranes thus represents an essential component of normal high-energy metabolism, with CT1 often recognized as a possible therapeutic target for the modulation of creatine homeostasis (3). This perspective paper explores several agents and vehicles that could switch CT1 upregulation and facilitate creatine uptake, and discusses the pros and cons of this strategy for experimental and clinical nutrition.

**Table 1:** Basic structural and functional characteristics of the creatine transporter.

	**Description**
Protein	Sodium- and chloride-dependent creatine transporter 1 (CT1)
Taxonomic ID	9606 (NCBI)
Gene^*^	Solute carrier family 6 member 8 (*SCL6A8*), locus Xq28
Organism	Homo sapiens
Size	635 amino acids (70.5 kDa); highly conserved (97%) between species
Subcellular location	Plasmalemma; possibly mitochondria
Tissue specificity	Skeletal muscle and kidney, brain, heart, colon, testis, prostate, etc.
Coupling ratio	2 Na^+^: 1 Cl^−^: 1 creatine
*K*_m_	15–77 μM
Sequences	4 Isoforms produced by alternative splicing

* *Several studies reported the existence of another human creatine transporter gene on chromosome 16p11.2; mRNA transcripts from this gene may only be expressed in the testis*.

## CT1 Modulation by Substrate Availability

The activity of creatine carriers appears to be partially regulated by the levels of intracellular creatine, with the amount perhaps modulating CT1 function by a feedback mechanism. The upregulation of CT1 expression by low creatine concentrations probably happens at the post-transcriptional level and may involve alternative splicing (4) and/or CT1 phosphorylation and glycolysation (5). A group from Columbia University was arguably the first who showed that creatine-starved myoblasts increased creatine transport activity for up to 3-fold above the levels observed in the cells maintained in a medium containing creatine (6). The authors reported that creatine must enter the cell to exert its regulatory activity on creatine transport by either regulating the number or turnover of CT1. Similarly, creatine uptake activity was significantly augmented in skeletal muscle membrane vesicles of rats who were subjected to 4-day creatine starvation (7) and in isolated hearts from creatine-free guanidinoacetate N-methyl transferase knockout mice (5), suggesting that fostering low creatine concentrations may upregulate CT1 and facilitate creatine assimilation. On the other hand, the creatine uptake can be reduced by the addition of exogenous creatine and consequent downregulation of CT1 expression, as seen in the skeletal muscle of rats supplemented with creatine for up to 6 months (8). Whether exposure to low creatine and concomitant upregulation of CT1 have any clinical potential remains unknown at the moment. Theoretically, if a creatine-free diet that instigates CT1 upregulation is followed by creatine loading, this could potentiate cellular uptake of creatine above normal amounts, a phenomenon that might be referred to as “creatine super-compensation.” This event is supposed to be transitory since the augmented intracellular creatine pool downregulates its own transport by 50% within 3–6 h (6).

## Stimulation of CT1 by KLOTHO Protein

Klotho protein (Clotho; HFTC3) exists in both full-length membrane form and soluble secreted form, playing a modulatory role in aging, bone metabolism, and endothelial dysfunction (9). Klotho, an enzyme and hormone, has been reported to participate in the regulation of cellular transport processes across the plasma membrane either indirectly through inhibiting calcitriol [1,25(OH)2D3] formation or other mechanisms or by directly affecting transporter proteins, including ion channels, cellular carriers, and Na(+)/K(+)-ATPase (10), and this might include CT1. The researchers from the University of Tübingen explored the effect of Klotho protein on CT1 modulation in the Xenopus oocyte experimental model (11). The authors found that the co-expression of Klotho protein increases a creatine-induced current in CT1-expressing oocytes, suggesting a Klotho-driven upregulation of creatine carriers, presumably by stabilizing the carrier protein in the cell membrane. The increase in creatine-induced current was reversed by a β-glucuronidase inhibitor (D-saccharic acid 1,4-lactone monohydrate), implying that upregulation of CT1 requires the β-glucuronidase activity of Klotho protein. In addition, Klotho protein levels required were within the range of concentrations encountered *in vivo*, which indicates that the stimulation of CT1 by Klotho likely exists in physiological conditions. Since Klotho protein can be activated by phosphate restriction, curcumin, or vitamin D [for a review see (12)], targeting the Klotho-CT1 axis by specific dietary interventions might therefore expedite creatine uptake and contribute to high-phosphate bioenergetics balance.

## Glucocorticoid-Inducible Kinases and CT1

The serum and glucocorticoid-regulated kinases are among the candidates involved in the regulation of CT1. These protein kinases are mainly expressed in the gut, brain, and endocrine tissues and play an important role in cellular stress responses by activating potassium, sodium, and chloride channels (13, 14). It appears that creatine transporter activity can be stimulated by glucocorticoid-inducible kinases in Xenopus oocytes heterologously expressing human CT1. Shojaiefard et al. (15) demonstrated that the serum and glucocorticoid-regulated kinases SGK1 and SGK3 stimulate CT1 by increasing the maximal transport rate of creatine through the carrier, an activity that may revive energy storage in myocytes and neurons. Tuning creatine uptake by SGK1 and SGK3 might happen due to ubiquitination, IGF-1-mediated pathway, osmolyte regulation, and/or phosphatidylinositol-3-phosphate-5-kinase activation (16), under both physiological and pathophysiological conditions. Kinetic analysis revealed that SGK1 enhanced the maximal current of creatine without significantly altering its affinity; the impact of SGK1 could be mimicked by the constitutively active isoform SGK3 but not by inactive SGK3. In terms of nutrition, 24-h starvation appears to display high levels of SGK1 in IIB fibers from the tibialis anterior (17), with SGK1 required to maintain pharyngeal muscle performance during starvation in *C. elegans* (18). Although CT1 expression and activity were not evaluated in these studies, triggering the serum and glucocorticoid-regulated kinases by food deprivation might be considered as yet another route for controlling the uptake of creatine.

## Protein Kinase MTOR and CT1

The mammalian target of rapamycin (mTOR) is a protein kinase that plays a major role in the regulation of cell growth, proliferation, and autophagy, while sensing cellular nutrient availability and energy levels. The mTOR pathway appears to be the central regulator of mammalian metabolism of tissues including the liver, skeletal muscle, adipose tissue, and brain (19), with the regulation perhaps including creatine metabolism. The group of Florian Lung from the University of Auckland demonstrated that mTOR affects creatine turnover by stimulating CT1 function (20), through mechanisms similar to the serum and glucocorticoid-inducible kinases. The authors found that co-expression of mTOR increased maximal creatine current through CT1 in Xenopus oocytes expressing bovine SLC6A8, while pre-incubation of the oocytes with rapamycin decreased the creatine-induced current and abrogated its stimulation by mTOR. Whether mTOR cross talks with SGK1 in the regulation of CT1 remains currently unknown, yet both kinases might participate in the adjustment of cellular creatine content to nutrient and energy supply (21). For instance, mTOR can be activated by various amino acids (22), time-of-day-dependent caloric restriction (23), or carbohydrate-restricted feeding (24), with diet-driven mTOR activation potentially followed by creatine stream *via* CT1.

## Hyperammonemia ElevateS CT1 Expression

Hyperammonemia is a metabolic condition characterized by the elevated levels of blood ammonia (4–150 times normal), leading to alterations in brain energy metabolism, blood–brain barrier dysfunction, and encephalopathy. Exposure to ammonium chloride (5 mM for 72 h, corresponding to pathophysiological levels observed in the brain in acute liver failure) resulted in a significant increase in mRNA levels of CT1 (1.9-fold increase) in conditionally immortalized mouse brain capillary endothelial cells (25). At the same time, the uptake of radiolabeled ^14^C-creatine was significantly increased by 18% in cells exposed to ammonia, possibly as a consequence of increased CT1 activity. The authors suggested that the augmented creatine transport across the blood–brain barrier in hyperammonemia could be implicated in neuroprotective mechanisms since creatine can afford significant neuroprotection (26). This is in line with Kosenko et al. (27), who reported that chronic hyperammonemia induced by a 20-day ammonium-containing diet ameliorated the clinical symptoms of acute ammonia intoxication and prevented the associated deficits in energy metabolism. Maintained levels of high-energy phosphates in the brain indicate that diet containing ammonium salts instigates adaptive alterations in energy metabolism that might be due to hyperammonemia-dependent upregulation of CT1. Still, creatine appears to be poorly taken up by immature embryonic brain cells in urea cycle defects that are accompanied by ammonia toxicity (28), suggesting a rather complex interconnection between hyperammonemia and creatine transport.

## Other CT1 Stimulants

Creatine accumulation *via* direct or indirect CT1 stimulation can be achieved by various hormones and hormone analogs (e.g., noradrenaline, isoproterenol, clenbuterol, 3,3′,5-triiodothyronine, amylin, growth hormone, insulin, insulin-like growth factor 1) (29–31). Schlattner et al. (32) reported an upregulation of CT1 after wounding of murine skin and increased abundance of creatine carriers in psoriatic human skin, leading to the accumulation of intracellular creatine. Pre-treatment with calyculin, a protein phosphatase 1a/2a inhibitor, abrogates the doxorubicin-induced creatine transport decrease (33), suggesting that CT1 stimulation is mediated by phosphorylation or a yet to be identified signal (34). CT1 expression and creatine uptake increase after adenoviral overexpression of peroxisome proliferator-activated receptor-γ coactivators 1a and 1b *via* estrogen-related receptor alpha (35), possibly identifying a new therapeutic gene target to increase intracellular creatine and tackle cellular energy homeostasis. A mechanistic nexus between diet and above CT1 excitants that might be involved in CT1 upregulation remains to be discovered.

## Possible Risks of CT1 Overexpression

Reduced levels of intracellular creatine critically imperil cellular bioenergetics, fostering CT1 upregulation and expedited creatine uptake. However, the cell appears to have an upper limit of creatine accumulation as well, implying a delicate balance between creatine levels and CT1 modulation on both sides of the coin. For instance, long-term creatine ingestion downregulates CT1 in order to prevent the excessive (and potentially harmful) intramuscular accrual of creatine (8). Wallis et al. (36) nicely demonstrated that the overexpression of CT1 in transgenic mice induces an excessive accumulation of creatine inside the myocytes, with an abnormally high intracellular creatine pool (66 ± 6 nmol/mg protein in wild-type controls vs. 133 ± 52 nmol/mg protein in CT1-overexpressing transgenic mice), accompanied by left ventricular dysfunction, myocardial hypertrophy, and heart failure. Likewise, mice overexpressing the myocardial CT1 experienced chronically increased levels of myocardial creatine and developed age-specific progressive hypertrophy and heart failure (37). Supra-normal myocardial creatine and phosphocreatine concentrations thus might lead to energetic impairment, probably due to the fact that the myocardium is incapable of keeping the augmented creatine pool adequately phosphorylated. On the other hand, Santacruz et al. (38) found no cardiac damage in mice with supraphysiological cardiac creatine levels. Adult transgenic animals showed an increase of 5.7-fold in the content of myocardial creatine, yet cardiac morphometry, echocardiography, and pressure–volume loop analyses demonstrated mild hypertrophy but normal function. Another trial suggested that mice overexpressing the creatine transporter in the heart (accompanied by the elevation of myocardial creatine by 20–100%) actually experienced a reduced myocardial stunning and ischemia/reperfusion injury (39), implying that increasing myocardial creatine for up to 100% was not detrimental but beneficial. Having this in mind, the magnitude of CT1 upregulation turns out to be of crucial importance for cell survival, since the maximum CT1 activity that can be attained without adverse metabolic effects is unknown at the moment. A risk-free ceiling for transporter function (along with maximal creatine levels) may vary from one cell type to another, requiring additional CT1 kinetics studies that address salient features encountered in creatine conveyance.

## Diet and CT1 Upregulation: Waiting in the Wings

Only a small number of *in vivo* studies reported the effects of controlled dietary regimens on CT1 upregulation, including a 4-day starvation test in male rats (7), a 6-month creatine-free diet in mice (5), and a 7-week creatine depletion feeding in rats (40). All regimens elicited a significant increase in creatine uptake and CT1 activity in the heart and skeletal muscle of experimental animals, likely due to an increased transporter protein expression mediated by low creatine concentrations (41). Those pilot studies were not followed by a torrent of pre-clinical studies and human trials probably due to the somewhat challenging quantification of CT1 expression, activity, and density in target cells (42). An interesting small-scale study observed lower muscle creatine levels and increased capacity to load creatine in seven vegetarian men (four vegans and three lacto-ovo vegetarians) who consumed a vegetarian diet for at least 6 months before the experiment (43). Muscle CT1 mRNA levels tended to be higher in vegetarians against non-vegetarian controls, which could partially explain an increased capacity to accumulate creatine in vegetarians subjected to creatine loading. Better control for diet composition in this pilot trial (i.e., the amount of creatine in vegan and lacto-ovo vegetarian nutrition has not been calculated) along with the inclusion of more participants would possibly reveal a more significant effect of creatine-free diet on CT1 upregulation. However, other possible mechanisms that rule out CT1 expression and density might be involved as well, including an accelerated maximal velocity of CT1, a reduced creatine efflux from the cell, or other unknown channels. An upregulation of CT1 gene expression and creatine deposition has been described in pigs and broilers who were supplemented with guanidinoacetic acid (GAA), a natural precursor of creatine (44, 45), yet the mechanism of GAA-driven CT1 stimulation remains unaddressed. Another nutritional study reported an elevated gene expression of CT1 in mice exposed to a 10-week high-fat diet and treated with nitrite (46), perhaps due to mechanisms that are both dependent and independent of proton-gradient uncoupling. Those exploratory studies lay the first stone of a possible role for diet in CT1 upregulation. This presumably complex tie-in urgently requires auxiliary research, including time-dependent changes in CT1 upregulation driven by a specific dietary regimen (e.g., acute vs. chronic effects of creatine-free diet), a food-driven CT1 triggering in various organs, stages of the life cycle and pathologies, and a possible synergism (or antagonism) of two or more food components to produce a combined effect on CT1 activity, to name just a few.

## Conclusion

**Figure 1:**
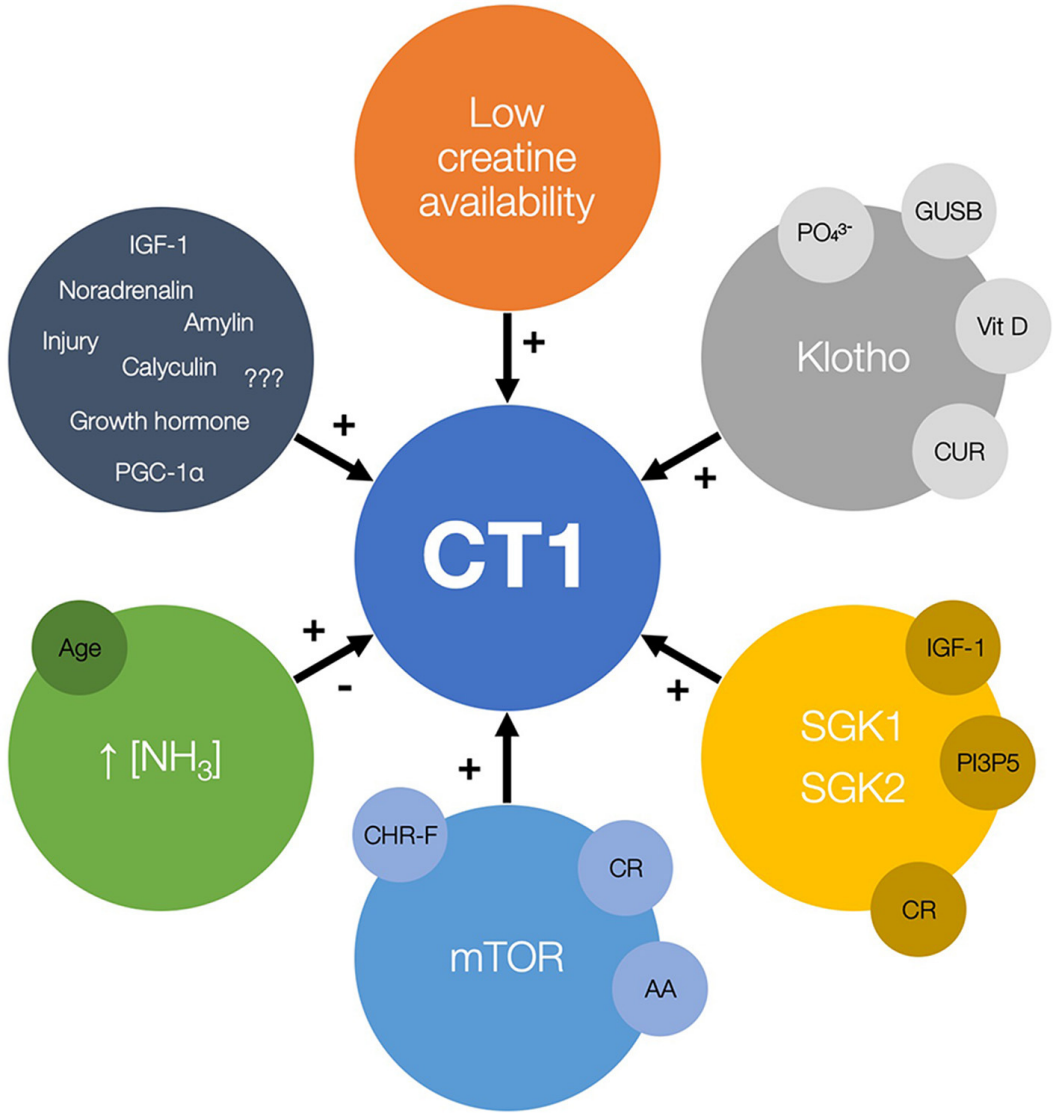
The candidate regulators of creatine transporter (CT1) function and expression, with possible nutrition-related cofactors and modulators (small circles). The plus sign (+) indicates the stimulation of CT1 activity while the minus sign (–) indicates possible inhibition of CT1 function. GUSB, β-glucuronidase; Vit D, vitamin D; CUR, curcumin; SGK, serum and glucocorticoid regulated kinases; IGF-1, insulin-like growth factor 1; PI3P5, phosphatidylinositol-3-phosphate-5-kinase; CR, calorie restriction; mTOR, mammalian target of rapamycin; AA, amino acids; CHR-F, carbohydrate-restricted feeding; PGC-1a, peroxisome proliferator-activated receptor-γ coactivators 1a.

Several vehicles are identified to upregulate or modulate CT1 function and uplift creatine allocation in a handful of *in vitro* and *in vivo* studies (Figure 1). Those include carrier modulation by low substrate availability, protein kinases, and hyperammonemia. Importantly, upregulation of CT1 also appears to be triggered by caloric restriction, creatine-free diet and exposure to ammonium-containing food. Upregulating CT1 could be therefore perceived as an up-and-coming target in nutritional sciences, yet its clinical efficacy, safety, and feasibility require a rather careful scrutinization in the forthcoming years.

## Author Contributions

The author confirms being the sole contributor of this work and has approved it for publication.

## Conflict of Interest

SO serves as a member of the Scientific Advisory Board on creatine in health and medicine (AlzChem LLC). SO owns patent “Sports Supplements Based on Liquid Creatine” at European Patent Office (WO2019150323 A1), and active patent application “Synergistic Creatine” at UK Intellectual Property Office (GB2012773.4). SO has served as a speaker at Abbott Nutrition, a consultant of Allied Beverages Adriatic and IMLEK, and an advisory board member for the University of Novi Sad School of Medicine, and has received research funding related to creatine from the Serbian Ministry of Education, Science, and Technological Development, Provincial Secretariat for Higher Education and Scientific Research, AlzChem GmbH, KW Pfannenschmidt GmbH, ThermoLife International LLC, and Monster Company. SO is an employee of the University of Novi Sad and does not own stocks and shares in any organization. The funders had no role in the design of the study; in the collection, analyses, or interpretation of data; in the writing of the manuscript, or in the decision to publish the results.
